# Recovery of Methanol during Natural Gas Dehydration Using Polymeric Membranes: Modeling of the Process

**DOI:** 10.3390/membranes12121176

**Published:** 2022-11-22

**Authors:** Daria Miroshnichenko, Vladimir Teplyakov, Maxim Shalygin

**Affiliations:** A.V. Topchiev Institute of Petrochemical Synthesis, Russian Academy of Sciences (TIPS RAS), 29 Leninskiy Prospect, Moscow 119991, Russia

**Keywords:** polymeric membranes, natural gas dehydration, membrane gas and vapor separation, methanol recovery, mass transfer modeling

## Abstract

A significant proportion of natural gas (NG) is produced in cold climates, where conditions are relevant to the formation of gas hydrates in raw gas stream. Methanol is often used as an effective inhibitor of hydrate formation. Further conditioning of NG includes dehydration, and the most common process of water vapor removal from NG is absorption. Absorption also provides removal of methanol vapors, which allows it reuse. The membrane method of natural gas dehydration is considered as a promising alternative; however, the study of methanol recovery by the membrane method, simultaneously to the dehydration of NG, has not been carried out previously. In addition, data on methanol vapor transfer in gas separation polymer membranes are almost absent in the literature. This paper evaluates the permeability coefficients of methanol vapors for several polymer materials, which are applied to the production of industrial membranes (PPO, PSf, CA). Mathematical modeling of the membrane process of NG dehydration with simultaneous recovery of methanol was performed. The dependencies of membrane area, methanol recovery and energy consumption for methane recycling and recompression on the process parameters are calculated. Obtained data show that the recovery of methanol during membrane dehydration of NG varies in the range 57–95%. The lowest values of membrane area and specific energy consumption were found for PPO based membrane.

## 1. Introduction

Natural gas (NG) is currently considered the cleanest traditional source of energy, and the proven reserves of NG exceed the reserves of coal and oil. Significant NG reserves are concentrated in the cold climatic regions of the north (Alaska, Canada, Russia) [1]. Natural gas streams from production wells are saturated with water vapor, which will condense, or can form gas hydrates if the gas temperature is cooled below its hydrate formation temperature. Gas hydrates are solids, which can agglomerate and plug pipelines and equipment, interrupting operations, stopping gas production and creating an unsafe condition.

One of the most effective thermodynamic inhibitor of hydrate formation is methanol. Inhibitors such as methanol are injected into the well or pipeline at the desired amount to ensure the operating condition is set outside the hydrate formation region [2]. Despite the development of low dosage hydrate inhibitors, the methanol is still widely used.

Condensed water in pipelines leads to erosion and corrosion. Water accumulation in the pipelines can lead to blockages and reduction in the pipeline flow capacity. To avoid these potential problems, the gas stream needs to be dried to lower its water dewpoint [3]. Depending on market specifications, the typical natural gas dew points cover the range from −5 to −20 °C as water dew point and from 0 to −10 °C as hydrocarbon dew point, while lower values can be required for subsea pipeline transportation.

Since the NG comes out of the well with a moisture content much higher than the specifications, its processing includes dehydration as an essential stage. The following NG dehydration technologies are used in industry: absorption, adsorption, low-temperature condensation, low-temperature separation, membrane separation, as well as combined methods. A comparison of the benefits and disadvantages of these technologies is given in Table 1.

Absorption with glycols (glycol dehydration) is the most common industrial technology for NG dehydration [4,5]. Glycol dehydration ensures the removal not only of water vapor but also of methanol vapor from the NG stream [6]. From the resulting water-methanol mixture, it is advisable to recover methanol for its reuse directly at the sites of extraction and preparation of NG.

Membranes offer an attractive option for cases in which drying is required to meet pipeline gas specifications and conventional glycol dehydration technologies are considered unfeasible due to the process’s equipment complexity. Benefits are expected to compare favorably to conventional technologies due to reduced energy consumption (absence of phase transition), no chemicals being required in the process, flexibility, easy integration with other processes and reduced emissions [7]. In this connection, research on NG membrane dehydration is continuously being conducted [8,9,10,11].

**Figure 1:**
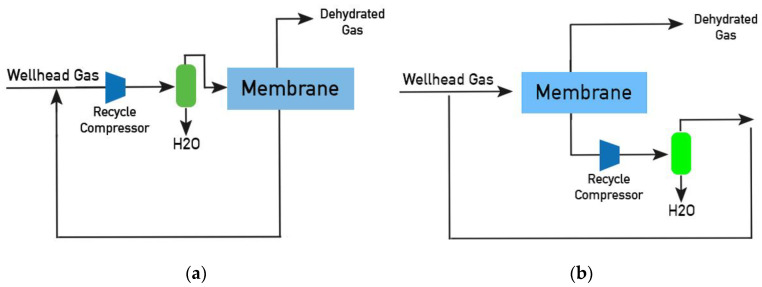
Air Liquide membrane process of NG dehydration [12]: (**a**) High pressure of wellhead gas; (**b**) Low pressure of wellhead gas.

For example, Air Liquide offers various options for the commercial implementation of the membrane process of NG dehydration using PEEK-Sep membrane [12]. In the case of high pressure NG (Figure 1a), it is fed into a membrane unit, where water vapor is removed through the membrane and a dehumidified gas is obtained in the retentate, the permeate is recompressed and, after separating the condensate, mixed with the initial flow. In the case of low NG pressure (Figure 1b), the flow is compressed, the condensate is separated at the separator, and the gas is fed into the membrane block; permeate from the membrane block is then mixed with the initial flow.

Modeling of separation processes significantly facilitates the selection of the most suitable technology and optimal parameters for a certain case. A significant number of studies regarding the modeling of NG membrane dehydration can be found. Some of the studies include evaluation of economic indicators for membrane dehydration and comparison with glycol dehydration of NG as a common technology. Results show that a membrane method has advantages under certain process parameters. For example, in [13] it is shown that the cost of membrane dehydration is lower under the following conditions: NG pressure is higher than 45 bar; the cost of the membrane is less than $ 165/m^2^; membrane permeance of water vapor is higher than 500 GPU. In [14], an economic assessment showed that membrane dehydration plants could be better value for money than the tri-ethylene glycol (TEG) depending only on predicted membrane lifetimes, on feed gas flow rates and on the operating conditions of the plant. Assuming the most likely membrane life span is 10 years, selective membrane systems can only claim to be the most cost-effective technology for offshore rigs of limited dimensions (processing up to 1,600,000 Sm^3^/day), while they definitely offer no economic advantage for medium to large rigs (over 3,250,000 Sm^3^/day). As for all the possible situations falling within these limit conditions, the advisability of installing a dehydration plant based on selective membrane modules needs to be evaluated case by case, in the light of the operating conditions that the system will be required to work with. A hybrid process of NG dehydration using MTR Pebax^®^ membrane and sweetening (removal of CO_2_) using carbon membrane was simulated in [15]. The distinguishing feature of the modelled process was that part of the captured CO_2_ was used as sweep gas in the dehydration unit to provide higher driving force for water permeation and reduce methane loss.

Studies on methanol extraction simultaneously with glycol dehydration of NG can be found in the literature; the degree of methanol extraction for installations of different capacities ranged from 40 to 70% of the total amount of methanol vapors in raw gas [16]. However, the studies of methanol transfer in the process of NG membrane dehydration were not performed, whereas the application of membrane dehydration for parallel recovery of methanol can introduce additional benefits for the process, including methanol reuse directly at the sites of NG extraction and preparation. The study of methanol recovery during membrane dehydration of NG is also complicated by the lack of data concerning the transfer of methanol in polymer membrane materials and membranes. Values of methanol permeability that were found for polydimethylsiloxane (PDMS) and poly (vinyl-trimethyl-silane) (PVTMS) are represented in Table 2.

This paper presents a theoretical study of NG membrane dehydration taking into account the presence of methanol vapors. Based on the available literature data of gas permeability and the application of the correlation approach [19], the methanol vapor permeability was estimated for a number of polymers, which are applied for the production of commercial gas separation membranes, namely poly (2,6-dimethyl-1,4-phenylene oxide) (PPO), cellulose acetate (CA) and poly-sulphone (PSf). Modeling of the NG membrane dehydration process with recovery of methanol was carried out. Characteristics of the separation such as methanol recovery, amount of methane recycling and specific energy consumption were determined depending on membrane used and on permeate pressure.

## 2. Methods

### 2.1. Estimation of Methanol Permeability in Polymeric Membranes

Gas transport in non-porous polymeric membranes is based on a solution-diffusion mechanism. The permeability coefficient is the product of solubility and diffusion coefficients [20]:

(1) P=DS

*P*–permeability coefficient (barrer), *D*–diffusion coefficient (cm^2^/s), *S*–sorption coefficient (cm^3^(STP)/(cm^3^∙cmHg)).

The correlation approach is based on statistically reasonable correlation equations of diffusion and solubility coefficients that were proposed based on comparative analysis of inert and permanent gases through a large number of polymers:

(2) $$\ln D=K_{0}^{D}+K_{1}^{D}d_{EF}^{2}$$

(3) $$\ln S=K_{0}^{S}+K_{1}^{S}{\left(\varepsilon /k\right)}_{EF}^{}$$

Taking into account Equation (1), the resulting equation for permeability coefficient comes as follows:

(4) $$\ln P=K_{0}^{D}+K_{0}^{S}+K_{1}^{D}d_{EF}^{2}+K_{1}^{S}{\left(\varepsilon /k\right)}_{EF}^{}$$

In case of absence of vapor influence on polymer, the transport of vapors also occurs by a solution-diffusion mechanism. Therefore, the correlation approach developed for estimation of gas transfer characteristics in polymers can also be used for estimation of vapor transfer characteristics.

For the determination of coefficients *K_i_* for a given polymer material, at least three known values of permeability coefficients of various gases in this polymer are necessary (it is not necessary to determine *K*_0_*^D^* and *K*_0_*^S^* separately as they can be reduced to single coefficient *K*_0_). In case of more data available than number of coefficients, the least squares method was used to solve the system, as in Equation (4). Parameters *d_EF_* and (*ε*/*k*)*_EF_* for gases are represented in [21]; for water and methanol molecules these parameters were determined in [18]. A set of data for membrane permeance can be used instead of permeability coefficient, that allows direct calculation of the permeance of membrane for a desired component. Permeability of methanol vapors was calculated for PPO, CA and PSf using literature data of gas and water vapor permeability: [22,23] for PPO, [22,23,24] for CA, [23,25] for PSf.

### 2.2. Modeling of NG Dehydration Membrane Process

**Figure 2:**
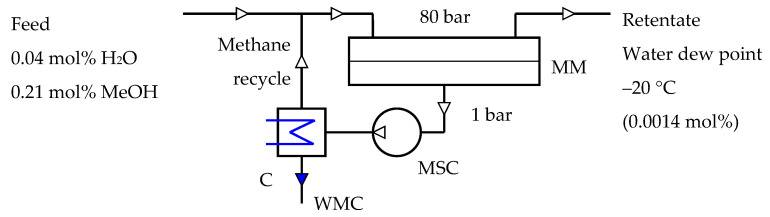
Scheme of membrane process of NG dehydration used for modeling (C–condenser, MSC–multistage compressor, MM–membrane module, WMC–water-methanol condensate).

**Figure 3:**
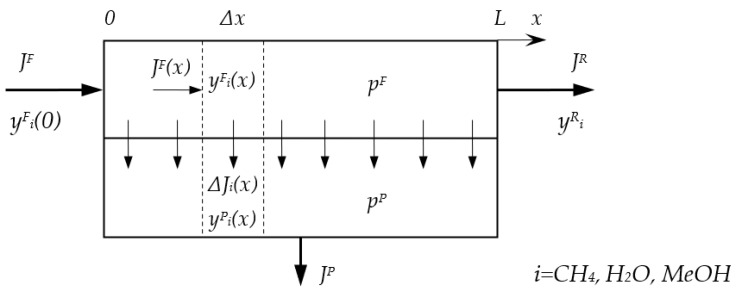
Scheme of mass transfer calculation in the membrane module used in the modeling.

Mathematical modeling was carried out for the one stage membrane process of NG dehydration shown in Figure 2. A basic model of gas transfer in membrane module, which operates in the cross-flow mode, was considered (Figure 3). The mathematical model includes the following assumptions: isothermal conditions; plug flow in feed membrane module channel; permeate drain from the membrane without mixing between surrounding regions; gas permeance is independent of feed gas composition and process conditions. Taking into account that values of methanol vapor permeability were estimated for several membranes, the consideration of other models (for example, counter-current model) and different effects (such as longitudinal pressure drop and mixing, concentration and temperature polarization, etc.) was not performed. These improvements in modeling will be more reasonable after experimental validation of methanol vapor permeability coefficients. Interstage cooling down to 30 °C was assumed during multistage compression of permeate.

Change of component flow in feed channel is determined by it passing through the membrane according to the equation:

(5) $$\Delta J_{i}^{F}(x)=-\Delta J_{i}^{P}(x)=-Q_{i}\Delta p_{i}(x)B\Delta x$$

Values of membrane permeance for components used in calculation were obtained using data from Table 2 and Table 4 and following thicknesses of selective layers: 3 μm for PDMS, 0.2 μm for PVTMS, 0.05 μm for PPO and PSf, 0.1 μm for CA. Driving force of a component transport through the membrane is determined by its partial pressure difference across the membrane:

(6) $$\Delta p_{i}(x)=p_{}^{F}y_{i}^{F}(x)-p_{}^{P}y_{i}^{P}(x)$$

Mole fraction of the component in the stream on the feed side of the membrane:

(7) $$y_{i}^{F}(x)=\frac{J_{i}^{F}(x)}{\sum_{i}J_{i}^{F}(x)}$$

Mole fraction of the component in the stream on the permeate side of the membrane:

(8) $$y_{i}^{P}(x)=\frac{J_{i}^{P}(x)}{\sum_{i}J_{i}^{P}(x)}$$

Incoming flows of components:

(9) $$J_{i}^{F}(0)=J_{}^{F}y_{i}^{F}(0)$$

Outcoming flows:

(10) $$J_{}^{P}=\sum_{i}^{}J_{i}^{P}$$

(11) $$J_{i}^{P}=\frac{1}{L}\int_{0}^{L}J_{i}^{P}(x)dx$$

(12) $$J^{R}=\sum_{i}J_{i}^{F}(L)$$

The system of equations was solved numerically using the finite difference method. The recovery of components was calculated as follows:

(13) $$\theta_{i}=\frac{J_{i}^{P}}{J_{i}^{F}}$$

The mathematical model is suitable for any membrane configuration (flat sheet, spiral wound, hollow fiber) where cross-flow approximation is realized. Modeling of single stage membrane dehydration of NG was carried out for the case of achieving water dew point in dried NG equal to −20 °C (0.0014 mol% H_2_O); other parameters used in calculation are listed in Table 3.

## 3. Results and Discussion

Values of methanol permeability in PPO, CA and PSf calculated using correlation approach are given in Table 4. Values of membrane permeances used for calculation are given in Table 5.

The validation of the mathematical model for NG membrane dehydration was carried out using data of [26], where a similar case (case “b”) of cross-flow membrane module operation for NG dehydration was considered. Initial data for calculation were: feed pressure 68.7 bar; permeate pressure 0.45 bar; feed flowrate 1.5 m^3^ (STP)/s; water content in feed 1000 ppm; water vapor permeance of membrane 1000 GPU; water/methane membrane selectivity 500; membrane area 280 m^2^. Obtained results have shown very similar values: 106 ppm of water in retentate compared to 100 ppm in reference work; 44,800 ppm of water in permeate compared to 45,000 ppm in reference work; permeate flow rate of 0.0300 m^3^ (STP)/s compared to 0.03 m^3^ (STP)/s in reference work.

**Figure 4:**
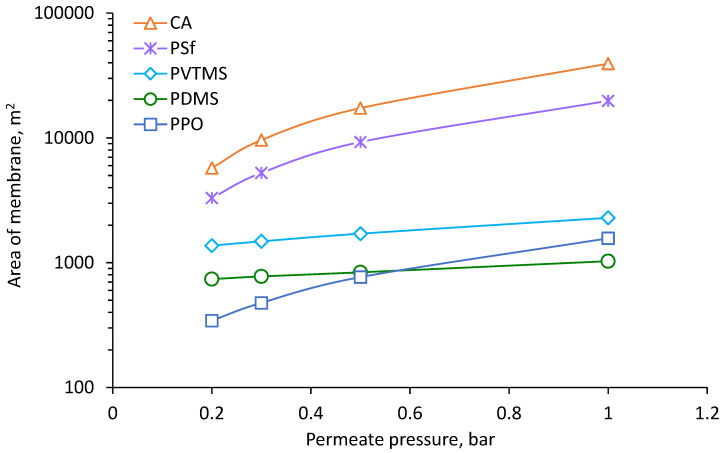
The dependence of the membrane area on the permeate pressure for achieving a water dew point of −20 °C.

**Figure 5:**
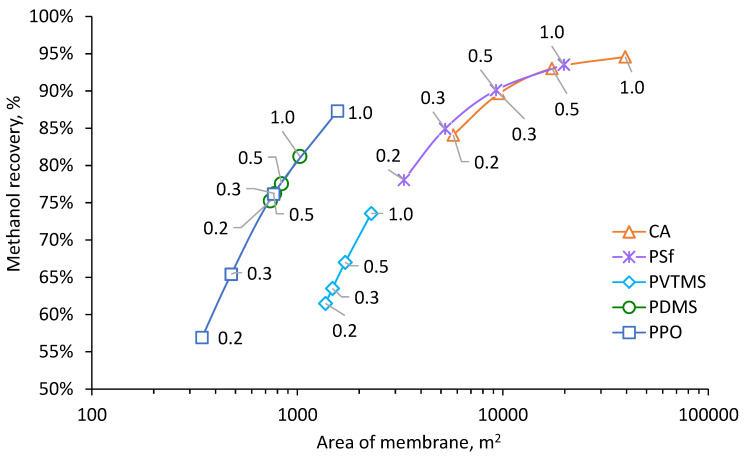
Dependence of methanol recovery on membrane area and permeate pressure for achieving a water dew point −20 °C.

**Figure 6:**
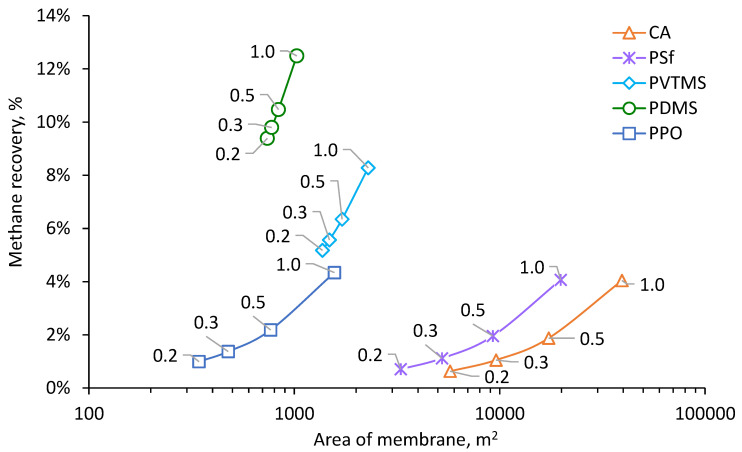
Dependence of methane recovery on the membrane area and permeate pressure for achieving a water dew point −20 °C.

**Figure 7:**
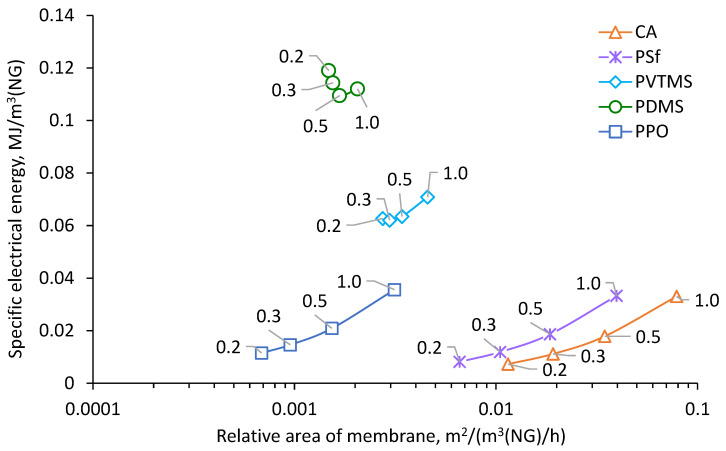
Dependence of specific electrical energy consumption for methane recompression on the relative area of membrane.

Results of calculation for the process of NG dehydration with removal of methanol vapor are shown in Figure 4, Figure 5, Figure 6 and Figure 7. The calculated membrane area, which is necessary for achieving a water dew point of −20 °C, depending on permeate pressure, is shown in Figure 4. Increasing of permeate pressure leads to increase of required membrane area since the driving force of water vapor transfer decreases. This dependence is much stronger for CA, PSf and PPO based membranes due to very high H_2_O/CH_4_ selectivity (see Table 4). Since partial pressure of water vapor in feed is much lower than absolute pressure of permeate, water vapor in permeate needs to be diluted by methane in order to achieve lower partial pressure than in feed and provide driving force for vapor transfer through the membrane. Increasing of permeate pressure demands more gas for dilution of water vapor and this leads to proportional increasing of the membrane area. The variation of area for PDMS and PVTMS based membranes is less sensitive to permeate pressure due to much lower H_2_O/CH_4_ selectivity. The highest membrane area is demanded for CA based membrane because of the lowest permeance of methane. The lowest values of demanded area were found for PDMS and PPO based membranes because of the combination of high methane permeance with high water permeance (Table 5); depending on permeate pressure, one or another membrane provides a lower area. The range of PPO based membrane area also intersects with the range of PVTMS based membrane area, which has intermediate values of methane and water permeance compared to PDMS and PPO. The reduction of permeate pressure leads to a decrease in capital costs depending on membrane area (number of modules). On the other hand, when the pressure is reduced, additional operating costs are required for extra energy of methane recompression.

The dependence of methanol recovery on membrane area is shown in Figure 5 with identifiers of corresponding permeate pressure. Results show that methanol recovery degree varies greatly in the range 57–95%, depending on the membrane used and permeate pressure; the highest values of almost 95% are achieved with CA and PSf based membranes.

A decrease of methanol recovery with reduction of permeate pressure is due to the decreasing membrane area required for achieving a given water dew point when permeate pressure decreases; thus, a smaller amount of methanol is extracted from the feed stream through the membrane. Close match of dependencies for PDMS and PPO based membranes is connected with close values of methanol permeance (see Table 5). For the same reason, dependencies for PVTMS, PSf and CA based membranes coincide with a common trend. The lowest methanol recovery degree was found to be equal to 57%, which can greatly improve its reuse in NG production.

Increase of membrane area increases not only methanol recovery but also methane recovery. Methane passed to permeate has to be recompressed for its return to the NG stream, which demands additional energy consumption. Obtained dependencies of methane recovery degree on the membrane used and permeate pressure are shown in Figure 6. The highest methane return (from 9–12%) is necessary for the case of PDMS membrane. CA, PSf and PPO membranes demonstrate almost the same methane recovery degree under the equal pressures of permeate.

The calculation of specific energy for methane recompression is performed in order to compare studied cases on the basis of relative capital and operating costs estimation. Figure 7 shows the relation of electrical energy and area of membrane normalized by NG feed flow rate. Reduction of specific electrical energy consumption with decrease of permeate pressure for PPO, PSf and CA membranes is interconnected with the influence of permeate pressure on membrane area. For these membranes, area is significantly decreased (by approximately five times) with reduction of permeate pressure, which leads to proportional reduction of permeate flow rate and demanded energy consumption. In case of PDMS and PVTMS membranes, the reduction of area is much weaker (about 25% and 40%, correspondingly) and reduction of permeate flow rate has less effect, thus at some point demanded energy for recompression becomes more significant and dependence begins to rise at lower pressure.

Obtained dependencies can be used as a basis for further calculation of economic indicators and selection of the optimal case. The cost of membrane area (membrane modules) and cost of electricity need to be set for a certain customer, as well as taking into account the cost of additional equipment and methanol reuse savings. The PPO based membrane can be considered as preferred method for the recovery of methanol in NG membrane dehydration.

## 4. Conclusions

Methanol permeability coefficients for PPO, CA and PSf were estimated on the basis of correlation approach and available data for gas permeability coefficients. Obtained data allowed mathematical modeling of NG dehydration by membrane separation with simultaneous recovery of methanol. Results show the variation of methanol recovery in the range of 57–95% depending on the membrane used and permeate pressure. Calculated dependencies demonstrate that PPO based membrane provides high recovery of methanol and low membrane area values in combination with low specific energy consumption compared to other membranes. PPO based membrane can be considered as preferred for the recovery of methanol in NG membrane dehydration.

## Figures and Tables

**Table 1:** Comparison of benefits and disadvantages of NG dehydration technologies.

Process	Benefits	Disadvantages
Absorption	Continuous process Using an inexpensive and readily available absorbent Low operating costs	High capital costs An absorbent regeneration stage is required Absorbent losses
Adsorption	Changes in temperature and pressure do not significantly affect dehydration quality Deep degree of purification (dew point −50 °C and below)	Frequency of the process High capital costs Reducing the capacity of the adsorbent and its degradation during operation (regeneration or replacement of the adsorbent) Loss of part of the product
Low-temperature separation	Ease of implementation Low capital and operating costs Absence of reagents Easy maintenance	Loss of gas flow pressure during throttling (subsequent compression is required) Dependence of the dehydration depth on the flow pressure
Low-temperature condensation	Absence of reagents Ease of implementation	High energy consumption to achieve low temperatures High capital costs for additional equipment
Membrane separation	Absence of phase transitions Absence of reagents Easy maintenance Modularity, compactness	Instability of some membranes in the presence of C_3+_ hydrocarbons Loss of a part of the product, or additional costs for compression and recycling

**Table 2:** Available data on methane, water and methanol permeability of membrane polymers.

Polymer	P(CH_4_), Barrer	P(H_2_O), Barrer	P(MeOH), Barrer	α(H_2_O/CH_4_)	α(MeOH/CH_4_)	Ref.
PDMS	950	36,000	13,900	38	15	[17]
PVTMS	18	760	230	42	13	[18]

**Table 3:** The simulation basis of a membrane process for natural gas dehydration.

Parameters	Values
NG feed flow rate, m^3^(STP)/h	500,000
Raw NG composition, mol%:	
CH_4_	99.75
H_2_O	0.04
MeOH	0.21
Feed pressure, bar	80
Permeate pressure, bar	0.2–1
Temperature, °C	25
Water dew point in dried NG, °C (mol%)	−20 (0.0014)
Compressor adiabatic efficiency, %	75
Number of recompression steps of permeate	3

**Table 4:** Available data on methane and water permeability and calculated values for methanol permeability in PPO, CA and PSf.

Polymer	P(CH_4_), Barrer	P(H_2_O), Barrer	P(MeOH), Barrer	α(H_2_O/CH_4_)	α(MeOH/CH_4_)	Ref.
PPO	2.3	4060	340 *	1765	147.8 *	[22,23]
CA	0.25	6000	161 *	24,000	644 *	[22,23,24]
PSf	0.25	2000	94 *	8000	376 *	[23,25]

* Calculated values.

**Table 5:** Permeances of membranes used in calculations.

Polymer Membrane	Selective Layer Thickness, µm	Q(CH_4_)∙10^6^, mol/(m^2^∙s∙kPa)	Q(H_2_O)∙10^6^, mol/(m^2^∙s∙kPa)	Q(MeOH)∙10^6^, mol/(m^2^∙s∙kPa)
PDMS	3	110	4100	1600
PVTMS	0.2	31	2300	570
PPO	0.05	22	28,000	2300
CA	0.1	0.84	20,000	550
PSf	0.05	1.7	14,000	640

## Data Availability

Not applicable.

## Nomenclature

*A*	membrane area, m^2^
*B*	width of membrane, m
*D*	diffusion coefficient, m^2^/s
*d_EF_*	effective diameter of gas during diffusion in polymer, m
*(ε/k)_EF_*	effective potential of gas interaction with polymer, K
*J*	flow rate, mol/s
*K*	coefficient, units depend on adjacent multiplier
*L*	length of membrane, m
*P*	permeability coefficient, barrer
*p*	pressure, bar
*Q*	permeance, mol/(m^2^∙s∙kPa)
*S*	sorption coefficient, mol/(m^3^∙kPa)
*x*	coordinate in membrane module, m
*y*	molar fraction, mol%
Greek letter	
α	selectivity of polymer/membrane, dimensionless
θ	recovery of component, %
Superscript	
D	diffusion
F	feed
P	permeate
R	retentate
S	sorption
Subscript	
0	free term of equation
1	first term of equation with an adjacent multiplier
i	component
